# Promotion of unproved and potential dangerous measures in fighting COVID-19 pandemic: urgent need for vigilant appropriate public communication and generation of scientific evidence

**DOI:** 10.11604/pamj.supp.2020.37.1.25547

**Published:** 2020-10-28

**Authors:** Florence Salvatory Kalabamu

**Affiliations:** 1 Hubert Kairuki Memorial University, Department of Pediatrics and Child Health, Dar es Salaam, Tanzania

**Keywords:** Misconception, COVID-19, evidence

## Abstract

The ultimate cure for COVID-19 has not yet been discovered, but there is a lot of promoted traditional and food supplements claimed to be effective against the disease. Some of the promoted measures are not only associated with other adverse health outcome, but also create a sense of false protection; leading to failure to follow appropriate measures. It is crucial to identify, correct this misinformation, and to conduct clinical trials to generate evidence among those which are scientifically sound.

## Commentary

Unprecedented COVID-19 pandemic has led many countries in chaos, with tremendous effect in public health, economy, education and social wellbeing. Since the first cases were reported in China in December 2019 to August 2020, The World Health Organization (WHO) as reported around 20.2 million confirmed cases and claimed 740,000 lives worldwide. Desperate situation led to desperate preventive measures, but some of these measures are completely rhetoric; they have not been scientifically proved; some could be even more fatal than COVID-19 itself. Unfortunately, some of the measures are promoted by highly regarded members of the communities; who have a good number of loyal followers. Despite the adversity the world is facing, it is crucial to advocate scientifically sound and evidence based treatment and interventions. Some recommended frequent drinking of alcoholic beverages to sanitize the mouth and throat which could lead to alcohol intoxication and encourage other irresponsible behavior associated with excessive alcohol use. Compiled data have shown that from December 2019 to April 2020, about 800 people died due to alcohol intoxication while 5,900 were hospitalized due to methanol poisoning. Among those with methanol poisoning, 60 lost their eye sight [1]. Surprisingly, while watching the international news, one could hear a public figure suggesting the use of petrol to sanitize the cloth face mast and one would go far to suggest the use of disinfectant injection as a treatment for COVID-19. Inhaling petrol fumes, can lead to dizziness, drowsiness, difficulty in breathing and addiction, while injected disinfectant can lead to multorgan failure. Some information from India, were circulating advising the public to consume cow dung and urine. Furthermore, some publications in Saudi Arabia were encouraging consuming the concoction of camel urine and lime as a proactive measure against COVID-19.

In some areas, people were advised to inhale steam from boiled herbal concoction. Although this method was being used historically in management of respiratory diseases, but there is no enough evidence on their safety and effectiveness. It can lead to inhalation burns, dehydration and electrolyte imbalance. It can also exacerbate pre-existing conditions such as asthma. There are some of spices and food supplements which have been promoted to be used as the preventive measure or treatment of COVID-19. Most of these have been proved to have positive effects in some disease conditions and enhancing body immunity against diseases, but these effects have been wrongly extrapolated to COVID-19. Oranges, lemon, black paper, garlic, and turmeric, alone or in combination have been publicly promoted to be used as prevention or treatment of COVID-19, but there is no evidence of their effectiveness. Vitamin C which is available in oranges, lemon and black paper has been suggested to reduce the severity and duration of common cold, but results from clinical trials are inconclusive [2]. No study has been conducted to prove this claim in novel coronavirus. Ginger (*Zingiber officinale*) is a spice which since mediaeval times has been used in traditional treatment of different ailments such as arthritis, diabetes, vomiting, cancer and other numerous conditions. It has been proved invitro to have anti-inflammatory and anti-cancer activities, but results from clinical trials have produced inconsistent and inconclusive results [3].

Turmeric (*Curcuma longa*) also has a share among the most promoted preventive measures against COVID-19. It is a common spice, and it has a long history as a medicinal plant in Asia. It´s active ingredient (carcuma) has been suggested to have effects against allergies, arthritis and neurodegenerative diseases. However, results from clinical trials have shown that it has low bioavailability and high doses are required to produce desirable response which is also associated with side effects [4]. *Garlic* (*Allium sativum*) has also been on the light and its market surged in most of African and Asian countries. Its active ingredient (diallylsulfide) which is the source of the pungent smell of garlic, is suggested to have effect on cancers cells, hypertension and fibrinolysis [5,6]. It is also believed to have platelet aggregation inhibition and anti-oxidative effects, but most of the studies are epidemiological and invitro which do not provide enough therapeutic evidence. Most of these foods, were massively promoted especially in African countries, with no clear guidance and precautions. In excessive use, they can cause unwanted effects such as gastritis, bleeding diathesis, and drug interaction among patients using other medications such as anti-hypertensive, anticoagulants and anticancer drugs.

Another micronutrient which has been promoted is vitamin D. It enhances innate and adaptive immunity and it has been proved to have anti-inflammatory properties. Although low blood levels of 25-hydroxylcalciferol has been associated with increased severity of seasonal influenza flue [7], its effect is yet to be proved in COVID-19. Zinc, which a trace mineral element has also been proved to play a role in immunity. It has been proved to reduce the duration and severity of common cold in adults at a higher dose [8], but its effect in COVID-19 is not yet ascertained. Vitamin A is also an immune enhancer; it has been associated with hastening immune response after vaccination and reduction of other common childhood diseases [9,10], but its role in prevention of COVID-19 is still not established. Although some of the promoted products are commonly used nutrients, and some have been proved to have effects in other viral respiration infections, it is inappropriate to assume that they will also be effective in prevention and treatment of COVID-19. Every pathogen has a unique mechanism of invading the body; mechanism to evade the immune system, and also the body defenses might be activated differently with different outcomes. Other promoted practice may lead to other health problems; the use of these unsubstantiated methods may create a sense of protection among the users and neglect to use other scientifically proved protective measures such as use of face mask, frequent handwashing with water and soap and social distancing.

## Conclusion

Well randomized controlled trials should be conducted to determine the safety and effectiveness of presumed safe promoted traditional and nutritional supplement so as to gather enough scientific evidence in fighting COVID-19. Scientist should be on the frontline to actively debunk misinformation and advocate scientifically proved public health interventions in fighting COVID-19.
